# A Vitamin Pattern Diet Is Associated with Decreased Risk of Gestational Diabetes Mellitus in Chinese Women: Results from a Case Control Study in Taiyuan, China

**DOI:** 10.1155/2019/5232308

**Published:** 2019-04-04

**Authors:** Qiong Chen, Yongliang Feng, Hailan Yang, Weiwei Wu, Ping Zhang, Keke Wang, Ying Wang, Jamie Ko, Jiaxin Shen, Lingling Guo, Feng Zhao, Wenqiong Du, Shouhang Ru, Suping Wang, Yawei Zhang

**Affiliations:** ^1^Department of Epidemiology, Shanxi Medical University School of Public Health, Taiyuan 030001, China; ^2^Office for Cancer Prevention and Research, Affiliated Cancer Hospital of Zhengzhou University/Henan Cancer Hospital, Zhengzhou 450008, China; ^3^Department of Obstetrics, The First Affiliated Hospital, Shanxi Medical University, Taiyuan 030001, China; ^4^Department of Chronic Disease Epidemiology, Yale School of Public Health, New Haven, 06520 CT, USA; ^5^Department of Surgery, Yale University School of Medicine, New Haven 06520, USA; ^6^Department of Environmental Health Sciences, Yale School of Public Health, New Haven, 06520 CT, USA

## Abstract

Identification of modifiable dietary factors, which are involved in the development of gestational diabetes mellitus (GDM), could inform strategies to prevent GDM. Therefore, we examined the dietary nutrient patterns and evaluated their relationship with GDM risk in a Chinese population using a case control study design. A total of 1,464 GDM cases and 8,092 non-GDM controls were included in the final analysis. Dietary intake was assessed using a 33-item food frequency questionnaire, and nutrients were estimated using the Chinese Standard Tables of Food Consumption. Dietary nutrient patterns were identified using factor analysis, and their associations with GDM risk were evaluated using unconditional logistic regression models adjusting for total energy intake, maternal age, high blood pressure, education, maternal body mass index (BMI), parity, and family history of diabetes. A “vitamin” nutrient pattern was characterized as the consumption of diet rich in vitamin A, carotene, vitamin B2, vitamin B6, vitamin C, dietary fiber, folate, calcium, and potassium. For every quartile increase in the vitamin factor score during one year prior to conception, the first trimester, and the second trimester of pregnancy, the GDM risk decreased by 9% (OR: 0.91, 95%CI: 0.86-0.96), 9% (OR: 0.91, 95%CI: 0.86-0.96), and 10% (OR: 0.90, 95%CI: 0.85-0.95), respectively. The significant reduced GDM risk was seen in women regardless of age and parity, and slightly stronger effect was found in women whose age ≤ 30 and women who are nulliparous across the three time periods. The significant association was also found in women whose BMI ≤ 24 with similar effect size across the three time periods. Our study suggests that the vitamin nutrient pattern diet is associated with decreased GDM risk. Additional studies are necessary to explore the underlying mechanism of this relationship.

## 1. Introduction

Gestational diabetes mellitus (GDM) is emerging as a public health concern due to the large disease burden and its increasing trend [1]. It affects approximately 5-17% of the pregnancies worldwide [2], and the prevalence has increased approximately 10-100% in several race/ethnicity during the past decades [3]. It would bring adverse consequences for GDM mothers and their offspring including cesarean delivery, shoulder dystocia, macrosomia, and neonatal hypoglycemia. In addition, women with GDM have a substantially increased risk to develop type 2 diabetes mellitus (T2DM) and cardiovascular disease (CVD) after pregnancy. Therefore, strategies for the effective prevention of GDM are mandatory.

Dietary components are associated with GDM development, making these modifiable factors ideal for informing GDM prevention strategies. Previous trials of early pregnancy dietary interventions (e.g., probiotic, myoinositol) have suggested beneficial effects on reducing GDM risk [4]. Observational studies also suggested that energy, nutrients (e.g., total fat, cholesterol, vitamin D, and heme iron), and food items (e.g., red/processed meats and eggs) were associated with risk of GDM [5]. Current evidence predominantly focused on single nutrient or food, the interactions or coeffects among the nutrients or foods were not considered.

Diet or nutrient pattern analysis would take the total effect of subjects' diet into account, and it has already become an advanced research hotspot. To date, most of the studies examining maternal dietary patterns and GDM had been conducted in Western population [5]. Adherence to the Mediterranean diet was found to be associated with decreased GDM risk or lower incidence of GDM in studies conducted in Iran [6], Mediterranean [7] and Australian [8] regions, and USA [9]. The Nurses' Health Study II found the Western dietary pattern [10] and low-carbohydrate dietary pattern [11] associated with elevated GDM risk. In Asian population, the Growing Up in Singapore towards Healthy Outcomes (GUSTO) study identified different diet pattern with that in Western populations and found that the seafood noodle-based diet was associated with a lower risk of GDM [12].

To the best of our knowledge, only one study including 3,063 pregnant women conducted in Guangzhou, China, examined dietary patterns in relation to GDM [13]. In this study, four dietary patterns, vegetable, protein-rich, prudent, and sweets/seafood, were identified. The highest tertile of vegetable score and sweets/seafood score was associated with reduced (OR = 0.79, 95%CI 0.64-0.97) and increased (OR = 1.23, 95%CI 1.02-1.49) risk of GDM, respectively. There are several limitations to this study. First of all, the study was conducted in Guangzhou, China. Given the vast differences in dietary habits between Northern and Southern China, the results of this study are not generalizable. Second of all, this study only used frequency of food intake to analyze dietary consumption and did not collect information on portion sizes, thus preventing the adjustment of total energy intake. Therefore, the effect of diet pattern and risk of GDM still needs to be verified in China. To examine the dietary pattern in Chinese population and evaluate its relationship with GDM risk, we conducted a case control study based on a birth cohort in Taiyuan, China.

## 2. Materials and Methods

### 2.1. Subjects

The subjects were recruited from a birth cohort at the First Affiliated Hospital of Shanxi Medical University in Taiyuan, China, which included 10,320 pregnant women waiting for delivery in the hospital during March 1, 2012, and December 30, 2016. Women aged 18 years or older with gestational age of 20 weeks or more and without mental illness were eligible for the study. In the birth cohort, 91 pregnant women with previous diabetes and 94 pregnant women whose gestational age is less than 24 weeks were excluded, resulting in a total sample size of 10,137 pregnant women.

All subjects gave their informed consent for inclusion before they participated in the study. The study was conducted in accordance with the Declaration of Helsinki, and the protocol was approved by the Ethics Committee of Shanxi Medical University. Information on demographic factors, reproductive and medical history, smoking, and alcohol were collected using standardized questionnaires [14] administered by trained interviewers. Information on birth outcomes and pregnancy complications were acquired by investigating the medical records.

### 2.2. Nutrient Assessment

Dietary intake was assessed using a validated 33-item food frequency questionnaire (FFQ) through an in-person interview at the hospital before/after delivery [14–16]. The 33 food items include cereals (rice, wheat flour, and coarse food grain), meats (pork, beef, mutton, poultry, fresh water fish, marine fish, shrimp, crab, and shell fish), dairy (milk, milk powder, and yogurt), eggs, bean products (soybean milk, tofu, kidney beans, and cowpeas), green leafy vegetables, other vegetables (cabbages, carrots, tomatoes, eggplants, potatoes, mushrooms, peppers, bamboo shoots, agarics, and garlic), alga (nori and kelp), pickles, nuts, and fruits.

Participants were asked to report the frequency and consumption of standard portion size for each food during the year before conception along with the first (1-13 weeks), second (14-27 weeks), and third (≥28 weeks) trimesters of pregnancy. We did not analyze the food intake for the third trimester because GDM diagnosis usually occurs before this time period. The reported frequency and portion size of each food item were converted to grams per day. Dietary nutrients were estimated from the frequency of consumption and portion size of food items using the Chinese Standard Tables of Food Consumption [17]. A total of 31 nutrients including energy, protein, fat, carbohydrate, dietary fiber, cholesterol, vitamin A, carotene, vitamin B1, vitamin B2, vitamin B6, vitamin B12, folate, niacin, vitamin C, vitamin E, alpha-tocopherol equivalent, calcium, phosphorus, potassium, sodium, magnesium, iron, zinc, selenium, copper, manganese, iodine, total fatty acid, choline, and biotin were estimated and included in the final analysis.

### 2.3. Cases and Controls Selection

Information on GDM diagnosis was extracted from the medical records. Individuals' blood glucose levels were examined using a 75 g oral glucose tolerance test (OGTT) during 24-28 weeks of gestation. Subjects were diagnosed as having GDM if they met at least one of the following criteria: (1) fasting blood glucose > 5.1 mmol/L, (2) 1-hour blood glucose > 10.0 mmol/L, and/or (3) 2-hour blood glucose > 8.5 mmol/L. A total of 1,523 women had GDM (cases) while 8,614 pregnant women did not (controls). Due to missing FFQ information, 59 cases and 522 control subjects were excluded from the study. In the end, a total of 1,464 cases and 8,092 control subjects were included in the analysis.

### 2.4. Statistical Analysis

All statistical analysis was conducted in SAS software (SAS Institute, Cary, NC). A chi-squared test was conducted to compare the difference of each characteristic between cases and controls. Nutrient patterns were estimated via principal component factor analysis. The factors were rotated by an orthogonal transformation (Varimax rotation function in SAS) to achieve simpler structure with greater interpretability. The eigenvalues, the Scree test, and the interpretability of factors were utilized to determine the number of factors. The factor loadings of the nutrient pattern were calculated, and a positive loading indicates a positive association with the factor, whereas a negative loading indicates an inverse association with the factor. The larger the loading of a given nutrient, the greater the contribution of the factor. The factor score of each factor generated by factor analysis was calculated from standardized scoring coefficients. The factor score for each pattern was regrouped into 4 groups according to quartile. A linear trend test was conducted by assigning the ordinal values 1, 2, 3, and 4 to Q1, Q2, Q3, and Q4, respectively. Unconditional logistic regressions were used to estimate odds ratios (ORs) and 95% confidence interval (95%CI) in order to determine the associations between nutrient patterns and GDM risk after adjusting for other factors identified by the factor analysis, total energy intake, maternal age, high blood pressure, education, maternal BMI, parity, and family history of diabetes.

## 3. Results

Compared to controls (Table 1), GDM cases were more likely to report a family history of diabetes (*P* < 0.0001), be older in age (*P* < 0.0001), have higher levels of education, have greater prepregnancy BMI (*P* < 0.0001), gain less weight during pregnancy (*P* < 0.0001), have high blood pressure during pregnancy (*P* < 0.0001), have higher parity, and have increased risk of preterm delivery (*P* = 0.020). Cases and controls had similar distributions of alcohol drinking and exposure to passive smoking during the first trimester.

A total of four patterns were generated in the factor analysis, and they could explain 89.73% of the variance of the original information. Only vitamin nutrient dietary pattern was included in the further analysis, while other three factors were not included in the analysis due to their lack of interpretation in the study area. The variance of vitamin nutrient dietary pattern was 12.63, and it accounted for 45.50% of total variance among the four factors. The factor loading of the vitamin nutrient dietary pattern during the previous year before conception and the first and second trimesters of pregnancy is shown in Table 2. A “vitamin” pattern was identified that loaded heavily with the following nutrients: vitamin A, carotene, vitamin B2, vitamin B6, folate, vitamin C, dietary fiber, protein, calcium, and phosphorus across the three time periods.

The contents of nutrients stratified by quartile of factor score for the “vitamin” nutrient pattern during the three periods as mentioned above are presented in Table 3. Most of the nutrients included in the vitamin pattern had higher contents in the last quartile (Q4) than in the first quartile (Q1); however, the content of carbohydrate was higher in Q1 than in Q4.

As shown in Table 4, vitamin dietary pattern was associated with a reduced risk of GDM. The reduced risk of GDM associated with vitamin dietary pattern was consistent across the three time periods with similar effect size (*P* for trend: <0.0001, <0.0001, and <0.0001, respectively); the GDM risk reduced for about 10% when the score increased by a quartile.

As shown in Tables 5 and 6, analysis stratified by maternal age and parity suggested that statistically significant association between the vitamin dietary pattern and GDM risk was found in women regardless of age and parity, and a slightly stronger effect was found in women who were 30 years old or younger and women who are nulliparous across the three time periods. When the association was stratified by BMI, the significant results were only found in women whose BMI ≤ 24 with similar effect size across the three time periods (Table 7).

## 4. Discussion

To our knowledge, a “vitamin” dietary nutrient pattern was identified for the first time in China, and it is associated with decreased GDM risk. The “vitamin” nutrient patterns identified in early pregnancy were consistent, and the most contributed nutrients to them were vitamin A, carotene, vitamins B2 and B6, vitamin C, and calcium, followed by potassium, dietary fiber, and folate. The associations between “vitamin” nutrient pattern and GDM risk were supported by evidence in previous studies, in which the nutrients included in the pattern were reported to be associated with GDM risk. Oxidative status and antioxidant capacity were reported to be involved in the pathogenesis of GDM. Nutrients including vitamins C and A were known to have antioxidant properties in vivo [18]. Low maternal dietary vitamin C intake and low plasma ascorbic acid were found to be associated with an increased risk of GDM in a case control [19] and cohort study [20]. Antioxidant capacity is lower in women with GDM, related to lower intake of vitamin E and zinc [21]; however, the difference of vitamin C and *β*-carotene was not found in women with and without GDM [21].

Dietary fiber diet was reported to be associated with GDM risk in previous studies [22], and the beneficial effect of high-fiber diets can be mainly attributed to soluble dietary fiber [23]. The potential mechanism of the beneficial effect of high-dietary fiber diet may be the improvement of insulin sensitivity, reduced adiposity, reduction of inflammatory markers, and alterations of various gut- and adipocyte-derived hormones [24] induced by it. Dietary fiber could reduce adiposity and improve insulin sensitivity by reducing appetite and food consumption, delaying gastric emptying, and slowing food digestion and absorption [22, 25]. It was also reported that dietary fiber could improve lipid homeostasis by the role of SCFA (short-chain fatty acids, SCFA) which derived from it [24, 26].

Calcium is another important component in the “vitamin” nutrient pattern, and its metabolism affected by vitamin D is associated with diabetes. Calcium plus vitamin D supplementation in women with GDM had beneficial effects on their metabolic profile [27]. In a prospective cohort study, higher level of periconceptional dietary calcium intake, particularly intake of calcium-rich low-fat dairy products and whole grains, is associated with lower GDM risk [28]. The underlying biological mechanisms may involve the regulation of intracellular calcium affecting both insulin sensitivity and insulin release [29, 30] as well as appetite regulation and related fat intake [31].

However, components of folate and potassium in the “vitamin” nutrient pattern are conversely associated with GDM risk. High potassium levels during the first half of pregnancy were reported to be associated with higher risk for the development of GDM [32]. Folic acid supplement intake in early pregnancy was also reported to increase the risk of GDM [33]. The underlying mechanisms may be associated with the imbalance between vitamin B12 and folate and the unmetabolized plasma folic acid. High folate status was reported to exaggerate the metabolic effects of vitamin B12 deficiency and worsen insulin resistance [34]. Unmetabolized plasma folic acid also has been reported to be related to decreased natural killer cell cytoactivity [35], which was reported to be involved in the pathogenesis of GDM. Hence, the presence of folate and potassium in the “vitamin” nutrient pattern would lower the effect of reduced GDM risk.

GDM is a public health concern due to its large disease burden; at the same time, the drug therapy for GDM women during pregnancy should be with caution. Therefore, preventive strategies of GDM are urgent. Until now, to our knowledge, several modifiable factors including diet and lifestyles were associated with GDM risk; however, the evidence from intervention studies are still lacking [4]. Currently, intervention with nutrients probiotic and myoinositol was found to reduce GDM risk [4]. Our study suggested that the “vitamin” nutrient pattern was associated with decreased risk of GD; it may provide clues for the prevention of GDM. Maternal age, BMI, and parity were known to be documented GDM risk factors; the effect of reduced GDM risk was found in women regardless of age and parity, and it was seen in women whose BMI ≤ 24. These results may help find the preventive strategies for GDM with more accuracy; however, they still need to be verified by additional studies.

There are several strengths and limitations in our study. In terms of strengths, the diagnosis of GDM in our study was obtained by investigating medical records that were based upon national guidelines of GDM diagnosis. This was likely to minimize potential disease misclassification. Another strength is that information on potential confounders was collected using a standardized questionnaire, thus allowing us to control these potential confounders. A limitation may be that self-reported dietary intake could have led to measurement errors, and the resulting misclassification of dietary intake may have weakened the detection of an association of specific nutrient patterns with GDM. The second limitation is that the subjects were enrolled from a hospital setting, potentially limiting the generalizability of the result. Thirdly, the 33-item food frequency questionnaire (FFQ) which assessed the dietary intake among subjects was not formally validated previously; however, the nutrient pattern and its relationship with GDM risk were conducted in our study which would minimize the influence on the relationship. Another limitation was the case control design which usually results in differential recall bias between cases and controls and overestimation of the effect size.

In conclusion, the “vitamin” nutrient pattern diet rich in vitamin A, carotene, vitamin B2, vitamin B6, vitamin C, dietary fiber, folate, calcium, and potassium is associated with a decreased GDM risk. Future studies preferably consisting of appropriately designed trials are necessary to verify the results and provide strong evidence to provide information about GDM prevention strategies.

## Figures and Tables

**Table 1:** Distribution of selected characteristics of case and control subjects.

Characteristics	GDM subjects (*N* = 1464)		Control subjects (*N* = 8092)		*P*
	Number	%	Number	%	
*Age (years)*					
<25	78	5.33	956	11.81	<0.0001
25-29	571	39.00	3692	45.63	
≥30	815	55.67	3444	42.56	
*Ever attend college*					
Yes	1,062	72.54	5550	68.59	0.003
No	402	27.46	2542	31.41	
*Body mass index (kg/m^2^)*					
<18.5	109	7.45	1153	14.25	<0.0001
18.5-24.9	974	66.53	5996	74.1	
25-30	318	21.72	821	10.15	
>30	63	4.3	122	1.51	
*Pregnancy weight gain (kg)*					
X ± s	30.39 ± 11.52		31.59 ± 10.46		<0.0001
*Gestational week*					
X ± s	38.43 ± 2.12		38.23 ± 1.97		0.0005
*Alcohol drinking during pregnancy*					
Yes	2	0.14	4	0.05	0.220
No	1,458	99.59	8,071	99.74	
Missing	4	0.27	17	0.21	
*Ever exposed to smoking during the first trimester*					
Yes	181	12.36	1,013	12.52	0.950
No	1,279	87.36	7,060	87.25	
Missing	4	0.27	19	0.23	
*Parity*					
Nulliparous	661	45.15	4,123	50.95	<0.0001
Parous	803	54.85	3,969	49.05	
*High blood pressure during pregnancy*					
Yes	265	18.1	1,061	13.11	<0.0001
No	1,199	81.9	7,031	86.89	
*Preterm when subjects were delivered*					
Yes	339	23.16	1,656	20.46	0.02
No	1,125	76.84	6,436	79.54	
*Family history of diabetes*					
Yes	193	13.18	432	5.34	<0.0001
No	1,271	86.82	7,660	94.66	

**Table 2:** Nutrient factor loadings during the three different periods of pregnancy.

Nutrients	One year before conception	The first trimester	The second trimester
Protein	0.21	0.24	0.23
Dietary fiber	0.28	0.30	0.28
Vitamin A	0.94	0.94	0.94
Carotene	0.96	0.94	0.94
Riboflavin (VitB2)	0.44	0.51	0.48
VitB6	0.22	0.24	0.22
Folate	0.29	0.30	0.30
Vitamin C	0.49	0.50	0.46
Calcium (Ca)	0.48	0.55	0.51
Potassium (K)	0.41	0.44	0.42

^∗^Absolute values < 0.20 were excluded from the table for simplicity.

**Table 3:** The nutrient content in diet among subjects stratified by the quartile of vitamin factor score ^∗^.

Nutrients	Cases		Controls	
	Q1	Q4	Q1	Q4
*One year before conception*				
Protein (g)	57.51 ± 22.25	67.02 ± 25.99	56.96 ± 22.3	64.2 ± 22.99
Dietary fiber (g)	26.5 ± 9.61	32.17 ± 10.56	26.7 ± 10.35	31.74 ± 10.86
Vitamins (mg)^∗∗^	85.03 ± 33.02	130.7 ± 36.55	86.44 ± 36.26	130.76 ± 37.94
Calcium (Ca) (mg)	328.14 ± 108.57	482.5 ± 167.08	334.29 ± 126.12	465.92 ± 151.8
Potassium (K) (mg)	1720.62 ± 574.62	2299.57 ± 731.4	1727.71 ± 617.12	2245.62 ± 686.01
*The first trimester*				
Protein (g)	58 ± 22.54	68.7 ± 25.88	57.71 ± 22.42	66.08 ± 23.93
Dietary fiber (g)	26.44 ± 9.76	32.68 ± 10.79	26.81 ± 10.13	32.04 ± 11.04
Vitamins (mg)^∗∗^	84.26 ± 33.03	133.69 ± 40.65	87.11 ± 34.92	132.1 ± 40.26
Calcium (Ca) (mg)	369.29 ± 115.58	569.11 ± 238.71	369.01 ± 133.04	552.13 ± 194.75
Potassium (K) (mg)	1779.47 ± 580.61	2412.75 ± 749.42	1786.55 ± 615.54	2354.61 ± 712.47
*The second trimester*				
Protein (g)	59.08 ± 22.56	69.1 ± 24.15	59.35 ± 22.7	67.48 ± 24.2
Dietary fiber (g)	26.78 ± 9.76	32.97 ± 10.76	27.75 ± 10.37	32.35 ± 10.97
Vitamins (mg)^∗∗^	85.66 ± 33.66	134.32 ± 45.5	90.81 ± 36.47	131.24 ± 38.15
Calcium (Ca) (mg)	375.24 ± 120.06	564.15 ± 216.39	378.6 ± 140.54	547.23 ± 194.33
Potassium (K) (mg)	1804.72 ± 580.85	2415.13 ± 728.93	1840.68 ± 626.16	2368.64 ± 728.67

^∗^For simplicity, only nutrient content in the first and last quartiles of vitamin pattern factor score is presented here. ^∗∗^Vitamins including vitamin A, carotene, vitamin B2, vitamin B6, vitamin C, and folate.

**Table 4:** Vitamin pattern and risk of gestational diabetes mellitus.

Nutrient pattern	One year before conception				The first trimester of pregnancy				The second trimester of pregnancy			
	Cases	Controls	OR^∗^	95%CI	Cases	Controls	OR^∗^	95%CI	Cases	Controls	OR^∗^	95%CI
*Vitamins*												
Q1	404	1,985	1.00		404	1,985	1.00		402	1,987	1.00	
Q2	378	2,011	0.92	0.78-1.08	369	2,020	0.89	0.76-1.04	388	2,001	0.95	0.81-1.11
Q3	371	2,018	0.86	0.73-1.01	380	2,009	0.89	0.76-1.04	355	2,029	0.81	0.69-0.95
Q4	311	2,078	0.71	0.61-0.84	311	2,078	0.71	0.60-0.84	319	2,075	0.74	0.63-0.87
Total	1464	8,092	0.90	0.85-0.95	1464	8092	0.90	0.86-0.95	1,464	8,092	0.90	0.85-0.95
*Ptrend*			**<0.0001**				**<0.0001**				**<0.0001**	

^∗^Adjusted for other factors, total energy intake, maternal age for years, high blood pressure, education, income, body mass index, parity, and family history of diabetes.

**Table 5:** Vitamin nutrient pattern and risk of GDM stratified by maternal age.

Vitamin pattern	Age ≤ 30				Age > 30			
	Cases	Controls	OR^∗^	95%CI	Cases	Controls	OR^∗^	95%CI
*One year before pregnancy*								
Q1	200	1,163	1.00		204	822	1.00	
Q2	166	1,149	0.80	0.63-1.00	212	862	1.04	0.83-1.30
Q3	142	1,142	0.67	0.53-0.85	229	876	1.06	0.85-1.32
Q4	141	1,194	0.65	0.51-0.82	170	884	0.78	0.62-0.98
Total			0.86	0.80-0.93			0.93	0.87-1.00
*Pfor trend*				**<0.0001**				0.054
*The first trimester*								
Q1	196	1,149	1.00		208	836	1.00	
Q2	155	1,159	0.73	0.58-0.92	214	861	1.05	0.84-1.31
Q3	153	1,155	0.72	0.57-0.91	227	854	1.07	0.86-1.33
Q4	145	1,185	0.67	0.53-0.85	166	893	0.74	0.59-0.94
Total			0.88	0.82-0.95			0.92	0.86-0.99
*Pfor trend*				**0.001**				**0.024**
*The second trimester*								
Q1	196	1,157	1.00		206	830	1.00	
Q2	169	1,162	0.81	0.65-1.02	219	839	1.09	0.87-1.36
Q3	135	1,146	0.65	0.51-0.82	220	883	0.99	0.79-1.23
Q4	149	1,183	0.70	0.56-0.89	170	892	0.76	0.61-0.96
Total			0.88	0.81-0.95			0.92	0.85-0.98
*Pfor trend*				**0.0006**				**0.016**

^∗^Adjusted for education, income, body mass index, parity, gestational week, high blood pressure, and family history of diabetes.

**Table 6:** Vitamin nutrient pattern and risk of GDM stratified by parity.

Vitamin pattern	Nulliparous				Parous			
	Cases	Controls	OR^∗^	95%CI	Cases	Controls	OR^∗^	95%CI
*One year before pregnancy*								
Q1	176	959	1.00		228	1,026	1.00	
Q2	178	1,063	0.92	0.73-1.16	200	948	0.92	0.74-1.14
Q3	167	1,049	0.83	0.65-1.05	204	969	0.89	0.72-1.11
Q4	140	1,052	0.70	0.55-0.90	171	1,026	0.73	0.58-0.91
Total			0.89	0.83-0.96			0.91	0.85-0.97
*Pfor trend*				**0.003**				**0.006**
*The first trimester*								
Q1	170	953	1.00		234	1,032	1.00	
Q2	176	1,055	0.91	0.72-1.15	193	965	0.87	0.70-1.08
Q3	174	1,057	0.86	0.68-1.09	206	952	0.92	0.74-1.13
Q4	141	1,058	0.70	0.55-0.90	170	1,020	0.71	0.57-0.89
Total			0.90	0.83-0.97			0.91	0.85-0.98
*Pfor trend*				**0.006**				**0.008**
*The second trimester*								
Q1	172	938	1.00		230	1,049	1.00	
Q2	189	1,068	0.94	0.75-1.18	199	933	0.95	0.76-1.18
Q3	153	1,051	0.74	0.58-0.95	202	978	0.88	0.71-1.09
Q4	147	1,066	0.72	0.56-0.91	172	1,009	0.76	0.60-0.94
Total			0.88	0.82-0.95			0.91	0.85-0.98
*Pfor trend*				**0.001**				**0.011**

^∗^Adjusted for maternal age for years, education, body mass index, gestational week, high blood pressure, and family history of diabetes.

**Table 7:** Vitamin nutrient pattern and risk of GDM stratified by BMI.

Vitamin pattern	BMI ≤ 24				BMI > 24			
	Cases	Controls	OR^∗^	95%CI	Cases	Controls	OR^∗^	95%CI
*One year before pregnancy*								
Q1	279	1,627	1.00		125	358	1.00	
Q2	261	1,676	0.92	0.76-1.11	117	335	0.93	0.68-1.26
Q3	246	1,644	0.85	0.71-1.03	125	374	0.87	0.65-1.18
Q4	190	1,687	0.66	0.54-0.81	121	391	0.81	0.60-1.10
Total			0.88	0.83-0.94			0.93	0.85-1.03
*Pfor trend*				**<0.0001**				0.158
*The first trimester*								
Q1	285	1,621	1.00		119	364	1.00	
Q2	250	1,673	0.84	0.69-1.01	119	347	1.05	0.77-1.43
Q3	249	1,643	0.84	0.70-1.01	131	366	1.03	0.76-1.39
Q4	192	1697	0.64	0.52-0.78	119	381	0.88	0.65-1.20
Total			0.88	0.82-0.93			0.96	0.87-1.06
*Pfor trend*				**<0.0001**				0.408
*The second trimester*								
Q1	283	1,615	1.00		119	372	1.00	
Q2	263	1,681	0.88	0.73-1.06	125	320	1.15	0.85-1.56
Q3	234	1,633	0.79	0.66-0.96	121	396	0.85	0.63-1.16
Q4	196	1,705	0.66	0.54-0.80	123	370	0.93	0.68-1.26
Total			0.87	0.82-0.93			0.95	0.86-1.04
*Pfor trend*				**<0.0001**				0.277

^∗^Adjusted for maternal age for years, education, parity, gestational week, high blood pressure, and family history of diabetes.

## Data Availability

The data used to support the findings of this study are included within the article.
